# Semi-quantitative evaluation of early phase ^99m^Tc-DPD scintigraphy in patients with suspected cardiac amyloidosis

**DOI:** 10.1007/s12149-026-02209-w

**Published:** 2026-04-16

**Authors:** Lukas Kessler, Leon Ferber, Christoph Rischpler, Stephan Settelmeier, Tugce Telli, Zohreh Varasteh, Clemens P. Spielvogel, Tim Hagenacker, Wolfgang P. Fendler, Alexander Carpinteiro, Lars Michel, Ken Herrmann, Tienush Rassaf, Miriam Sraieb, David Kersting

**Affiliations:** 1 Department of Nuclear Medicine, University Hospital Essen, University of Duisburg-Essen, 45147 Essen, Germany; 2 West German Amyloidosis Center, Hufelandstr. 55, 45147 Essen, Germany; 3 Department of Cardiology and Vascular Medicine, West German Heart and Vascular Center, University Hospital Essen, Hufelandstrasse 55, 45147 Essen, Germany; 4 Division of Nuclear Medicine, Department of Biomedical Imaging and Image-guided Therapy, Medical University of Vienna, Vienna, Austria; 5 Comprehensive Center for Artificial Intelligence in Medicine, Medical University of Vienna, Vienna, Austria; 6 Department of Neurology, Center for Translational Neuro- and Behavioral Sciences (C-TNBS), University Medicine Essen, Hufelandstr. 55, 45147 Essen, Germany; 7 Department of Hematology, West German Tumor Center, University Hospital Essen, Hufelandstrasse 55, 45147 Essen, Germany; 8 Department of Molecular Biology, University of Duisburg-Essen, Hufelandstrasse 55, 45147 Essen, Germany; 9 Department of Nuclear Medicine, Klinikum Stuttgart, Prießnitzweg 24, 70374 Stuttgart, Germany; 10 Strahleninstitut Köln, Domstraße 49-53, 50668 Köln, Germany

**Keywords:** Cardiac amyloidosis, Transthyretin, ATTR, Imaging, DPD scintigraphy

## Abstract

**Background:**

Scintigraphy using bone-avid tracers like ^99m^Tc-diphosphono-propanodicarboxylic acid (^99m^Tc-DPD), ^99m^Tc-hydroxymethylene-diphosphonate (^99m^Tc-HMDP), or ^99m^Tc-pyrophosphate (^99m^Tc-PYP) is a widely available and non-invasive imaging tool for diagnosis of cardiac amyloidosis. Visual evaluation and semi-quantitative image analyses reach excellent sensitivity and specificity results. For ^99m^Tc-HMDP, high accuracy of early semi-quantitative parameters derived from imaging 10 min after tracer injection was described but never validated for other tracers. The aim of this study was to evaluate early semi-quantitative parameters in ^99m^Tc-DPD scintigraphy.

**Methods:**

122 patients (78 male / 44 female) with suspected cardiac amyloidosis who underwent ^99m^Tc-DPD scintigraphy were retrospectively analysed. Early and late planar whole-body scintigraphy images were acquired 10 min (+ 5 min) and 2.5–3.5 h after tracer injection. Early cardiac ^99m^Tc-DPD uptake was evaluated using the Heart/whole-body rectangular (H/WBr), Heart/whole-body profile (H/WBp), Heart/Skull (H/S), Heart/Mediastinum (H/M), Heart/Contralateral Lung (H/CL), Heart/Liver (H/L), and Heart/Pelvis (H/P) ratios.

****Results**:**

H/L was not correlated to the other parameters.Best results for prediction of Perugini ≥1 were achieved for H/M (sensitivity 69.6%, specificity 84.4%, accuracy 77.5%), best results for prediction of ATTR-CA for H/L (sensitivity 65.9%, specificity 88.6%, accuracy 80.8%). Adding the H/L to other parameters increased accuracy and specificity (best results for H/L+H/S: accuracy 83.3%, sensitivity 63.4%, specificity 93.7% to predict CA).

****Conclusion**:**

Previous results for^99m^Tc-HMDP were not confirmed; diagnostic precision of early ^99m^Tc-DPD is not sufficient to replace late imaging in clinical routine. Early H/L is a novel, independent, and specific parameter which may be added to imaging protocols to increase the accuracy in combination with other imaging biomarkers

**Supplementary Information:**

The online version contains supplementary material available at 10.1007/s12149-026-02209-w.

## Introduction

Cardiac amyloidosis (CA) is a clinically long underestimated disease whose pathogenesis is characterized by myocardial deposition of different types of amyloid fibrils [1, 2]. However, in recent years, CA has been identified as a common cause of heart failure with preserved ejection fraction (HFpEF), with a high prevalence in HFpEF patients ≥ 75 years of age [3]. The most common types of systemic amyloidosis with cardiac involvement are transthyretin amyloidosis (ATTR) and immunoglobulin light chain amyloidosis (AL).

The gold standard of diagnosing CA is invasive endomyocardial biopsy. However, in current diagnostic work-up concepts it is often be replaced by non-invasive imaging [4]. A commonly applied method is scintigraphy using the bone-avid radiotracers ^99m^Tc-diphosphono-propanodicarboxylic acid (^99m^Tc-DPD), ^99m^Tc-hydroxymethylene-diphosphonate acid (^99m^Tc-HMDP), or ^99m^Tc-pyrophosphate (^99m^Tc-PYP) [5–8]. In the interpretation, cardiac tracer accumulation is visually assessed in comparison to bone uptake using the Perugini score [9]. Following the commonly applied Gillmore criteria, ATTR-CA can be diagnosed by a Perugini score ≥ 2 and absence of monoclonal protein in serum/urine sampling with high accuracy [10], particularly in advanced-stage patients. An additional advantage of whole-body imaging is the detection of extracardiac manifestations. However, scintigraphy-based diagnosis has not yet been sufficiently investigated in early stages of disease and clinically equivocal findings still demand histopathological validation in a relevant fraction of patients. Therefore, improved image interpretation is still an open clinical task.

An alternative to visual evaluation is semiquantitative image analysis that calculates ratios of cardiac tracer uptake to different reference regions. Using this approach, accurate diagnosis of cardiac amyloidosis and imaging-based sub-differentiation between ATTR- and AL-CA have been described. In planar imaging, semiquantitative indices such as H/CL, H/M, and H/WB have been proposed, primarily in the context of late-phase imaging [11, 12]. Typically, planar image acquisition is performed 3 h after tracer injection [9]. However, emerging evidence suggests that early-phase imaging may also provide clinically relevant information. Galat et al. [13] reported excellent performance of an early H/M derived from ^99m^Tc-HMDP images acquired shortly after injection for predicting late cardiac radiotracer accumulation (Perugini score) and discriminating ATTR-CA from AL-CA. Furthermore, in a tracer comparison study, Abulizi et al. reported comparable early-phase myocardial uptake intensity for ^99m^Tc-HMDP and ^99m^Tc-DPD in hereditary transthyretin-related cardiac amyloidosis [14], supporting the rationale to evaluate early-phase imaging using ^99m^Tc-DPD. Early imaging shortly after injection would be beneficial for both patient comfort and logistics.

To the best of our knowledge, early-phase imaging has, thus far, not been evaluated in a large clinical patient cohort using ^99m^Tc-DPD. Therefore, the aim of this study was to evaluate the accuracy of semiquantitative early-phase ^99m^Tc-DPD scintigraphy for prediction of late-phase Perugini scoring and diagnosis of CA. Moreover, combinations of different parameters were tested and compared to standard semiquantitative evaluations.

## Methods

### Patient population

122 patients (78 male/44 female) with suspected cardiac amyloidosis who underwent ^99m^Tc-DPD scintigraphy at University hospital Essen from June 2018 to June 2021 were included. All patients gave written informed consent to the examination. Data were retrospectively analysed and pseudonymised prior to evaluation. The study was conducted in accordance with the Declaration of Helsinki and approved by the local institutional ethics committee (University of Duisburg-Essen, medical faculty, Ethics protocol number 25–12485-BO).

### Image acquisition and evaluation of late ^99m^Tc-DPD images

A mean±standard deviation (SD) of 522.3 ± 26.1 MBq of ^99m^Tc-DPD was intravenously administered. Motivated by data from Galat et al. [11], early planar whole-body scintigraphy was performed approximately 10 min post-injection (allowable deviation + 5 min) using a Symbia S or Symbia Intevo gamma camera (both Siemens Healthineers, Erlangen, Germany). Late whole-body scintigraphy images were acquired 2.5–3.5 h after tracer injection according to our clinical routine protocol. Planar imaging was performed using a low energy high resolution collimator, a 256 × 1024 matrix, a whole-body scan speed of 15 cm/min, and a 140 keV ± 10% energy window centered on the ^99m^Tc photopeak. Identical acquisition parameters were applied for early and late imaging. Late images were evaluated independently by two experienced nuclear medicine physicians (L.K.; D.K.) to determine the Perugini score [9].

### Diagnosis of cardiac amyloidosis

Diagnosis of AL-CA and ATTR-CA was performed by endomyocardial biopsy. If endomyocardial biopsy was not available, imaging-based diagnosis was applied (only for ATTR-CA). Following the Gillmore criteria [10], positive scintigraphy (Perugini ≥ 2) and absence of monoclonal protein (in protein electrophoresis, serum free light-chain assay, urine/blood immunofixation) were required for diagnosis of ATTR-CA. Extracardiac tissue biopsy reports were evaluated if available.

### Semiquantitative analysis of early ^99m^Tc-DPD images

Images were analysed using the MM Reading workflow of Syngo.via (Version VB80A; Siemens Healthineers, Erlangen, Germany). Early cardiac ^99m^Tc-DPD uptake was evaluated using the following semiquantitative indices: Heart/whole-body rectangular (H/WBr), Heart/whole-body profile (H/WBp), Heart/Skull (H/S), Heart/Mediastinum (H/M), Heart/Contralateral Lung (H/CL), Heart/Liver (H/L), and Heart/Pelvis (H/P). H/WBr, H/WBp, H/S, H/CL, and H/P were motivated by previous publications for late imaging [12], and H/M by a previous publication for early ^99m^Tc-HMDP scintigraphy [11]. For all indices, cardiac tracer uptake was evaluated in a heart region of interest (ROI) and normalised by different reference ROIs. In early planar imaging, myocardial uptake cannot be reliably separated from ventricular blood-pool activity; therefore, the cardiac ROI should be interpreted as reflecting whole-heart uptake including blood-pool contribution. ROIs were placed by two experienced readers (L.K.; D.K.) in consensus using a standardized approach, and early semiquantitative analyses were performed in a separate reading session blinded to the clinical diagnosis and to late-phase Perugini scores. Semiquantitative indices were calculated using the following definitions as previously published by Gallini et al. [12]:

- **Heart/whole-body rectangular ROI ratio (H/WBr)**: Cardiac uptake was normalised by uptake in a rectangular whole-body ROI. A rectangular ROI embracing the patient body and three additional anatomical ROIs (bladder and the two kidneys) were placed to evaluate the tracer uptake. The rectangular ROI was chosen as a clinically practicable, robust approach to assess whole body uptake. The H/WBr uptake ratio was defined as:

$$\:{\mathrm{H}}/{\mathrm{WBr}}\: = \:\frac{{{\mathrm{Heart}}}}{\begin{gathered} ({\mathrm{Wholebody}}\:{\mathrm{rectangular}}\: - \:{\mathrm{bladder}}\: \hfill \\ - \:{\mathrm{left}}\:{\mathrm{kidney}}\: - \:{\mathrm{right}}\:{\mathrm{kidney}}) \hfill \\ \end{gathered} }$$

- **Heart/whole-body profile ROI ratio (H/WBp)**: Cardiac uptake was normalised by uptake in a profile whole-body contour ROI following the shape of the patient body. An anatomical contour ROI embracing the patient body and three additional anatomical ROIs (bladder and the two kidneys) were placed to evaluate the tracer uptake. The H/WBp uptake ratio was defined as:

$$\:{\mathrm{H}}/{\mathrm{WBp}}\: = \:\frac{{{\mathrm{Heart}}}}{\begin{gathered} ({\mathrm{Wholebody}}\:{\mathrm{profile}}\: - \:{\mathrm{bladder}}\: \hfill \\ - \:{\mathrm{left}}\:{\mathrm{kidney}}\: - \:{\mathrm{right}}\:{\mathrm{kidney}}) \hfill \\ \end{gathered} }$$

- **Heart/Skull ratio (H/S)**: Cardiac uptake was normalised by uptake in a skull contour ROI following the anatomical shape of the skull. The H/S uptake ratio was defined as:

$$\:\mathrm{H}/\mathrm{S}=\:\frac{\mathrm{H}\mathrm{e}\mathrm{a}\mathrm{r}\mathrm{t}}{\mathrm{S}\mathrm{k}\mathrm{u}\mathrm{l}\mathrm{I}}$$

- **Heart/Mediastinum ratio (H/M)**: Cardiac uptake was normalised by uptake in an oval ROI placed in the upper mediastinum. The H/M uptake ratio was defined as:

$$\:\mathrm{H}/\mathrm{M}=\:\frac{\mathrm{H}\mathrm{e}\mathrm{a}\mathrm{r}\mathrm{t}}{\mathrm{M}\mathrm{e}\mathrm{d}\mathrm{i}\mathrm{a}\mathrm{s}\mathrm{t}\mathrm{i}\mathrm{n}\mathrm{u}\mathrm{m}}$$

- **Heart/Contralateral Lung ratio (H/CL)**: Cardiac uptake was normalised by uptake in a contralateral lung contour ROI following the anatomical shape of the respective lung. The H/CL uptake ratio was defined as:

$$\:\mathrm{H}/\mathrm{C}\mathrm{L}=\:\frac{\mathrm{H}\mathrm{e}\mathrm{a}\mathrm{r}\mathrm{t}}{\mathrm{C}\mathrm{o}\mathrm{n}\mathrm{t}\mathrm{r}\mathrm{a}\mathrm{l}\mathrm{a}\mathrm{t}\mathrm{e}\mathrm{r}\mathrm{a}\mathrm{l}\:\mathrm{L}\mathrm{u}\mathrm{n}\mathrm{g}}$$

- **Heart/Liver ratio (H/L)**: Cardiac uptake was normalised by uptake in a liver contour ROI following the anatomical shape of the liver. The H/L uptake ratio was defined as:

$$\:\mathrm{H}/\mathrm{L}=\:\frac{\mathrm{H}\mathrm{e}\mathrm{a}\mathrm{r}\mathrm{t}}{\mathrm{L}\mathrm{i}\mathrm{v}\mathrm{e}\mathrm{r}}$$

- **Heart/Pelvis ratio (H/P)**: Cardiac uptake was normalised by uptake in an oval ROI placed in the pelvis. The H/P uptake ratio was defined as:

$$\:\mathrm{H}/\mathrm{P}=\:\frac{\mathrm{H}\mathrm{e}\mathrm{a}\mathrm{r}\mathrm{t}}{\mathrm{P}\mathrm{e}\mathrm{l}\mathrm{v}\mathrm{i}\mathrm{s}}$$

**Fig. 1:**
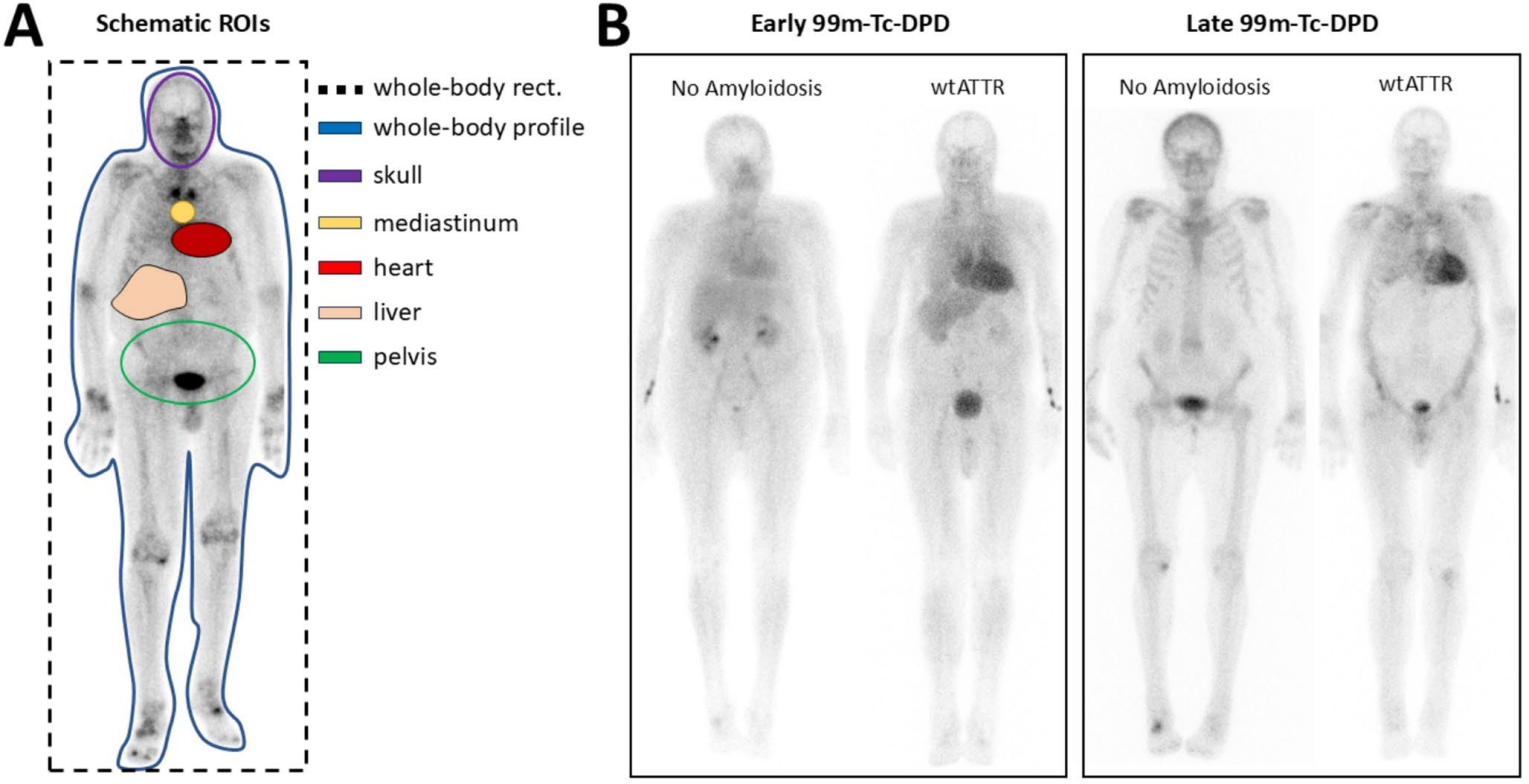
(**A**) Schematic delineation of respective ROIs in anterior projection of early-phase planar images, ROIs were similarly delineated in posterior projection (not shown). (**B**) Case examples of a patient with physiological tracer distribution in early phase (10 min p.i.) and Perugini 0 in late phase (2.5–3.5 h p.i.) 99 m-Tc-DPD scintigraphy compared to a patient with wtATTR amyloidosis and increased cardiac tracer uptake in early and late scintigraphy (Perugini 3)

Figure 1A shows the positions of the ROIs that were evaluated to calculate the semiquantitative indices. For comparison, all scores were separately calculated using anterior uniplanar images, the arithmetic mean of anterior and posterior images, and the geometric mean of anterior and posterior images. For all ratios, uptake values were estimated as mean values (total number of counts per ROI divided by total number of pixels per ROI). For H/WBr and H/WBp, in which kidneys and bladder were excluded from the whole-body ROI to correct for their variable uptake behaviour, mean whole-body uptake was calculated using the following definition:

$$\:mean\:WB = \:\frac{\begin{gathered} total\:counts\:WB\: - \:total\:counts\:bladder\: \hfill \\ - \:total\:counts\:right\:kidney\: \hfill \\ - \:total\:counts\:left\:kidney \hfill \\ \end{gathered} }{\begin{gathered} (total\:pixels\:WB\: - \:total\:pixels\:bladder \hfill \\ \: - \:total\:pixels\:right\:kidney\: \hfill \\ - \:total\:pixels\:left\:kidney) \hfill \\ \end{gathered} }$$

### Statistics

Correlations between early semiquantitative scores were evaluated using Spearman’s rho. To investigate the relationship between early semiquantitative scores as well as baseline demographic and imaging parameters and late Perugini scores, multivariate ANOVA (MANOVA) followed by Scheffé tests for post-hoc analyses were performed. For all statistical evaluations, a p-value < 0.05 was considered significant. Receiver operating characteristic (ROC) analyses were performed to assess the performance of early semiquantitative scores in predicting late Perugini scoring (Perugini 0 vs. 1–3 and Perugini 0–1 vs. 2–3) and in predicting ATTR-CA. Area-under-the-curve (AUC), accuracy, sensitivity, specificity, positive predictive value (PPV), and negative predictive value (NPV) were calculated, and optimal cut-off values were determined using Youden’s J.

To evaluate the diagnostic performance of combined imaging indices in predicting Perugini scores and the presence of cardiac amyloidosis, a sequential dual-threshold optimization approach was employed. For each combination of a primary index (H/WBp, H/S, H/M, or H/CL) and a secondary index (H/L), thresholds were determined in two stages. First, an initial cut-off for the primary index was established by optimizing diagnostic yield while maintaining a fixed specificity constraint of 90%. Second, in the subset of patients who met the primary threshold, a second cut-off for the secondary index (H/L) was optimized to maximize performance while maintaining a sensitivity constraint of 90%. AUC for each combination was calculated using logistic regression models incorporating both indices. Accuracy, sensitivity, specificity, PPV and NPV were derived from the final classification results. The analysis was performed on the total cohort and subsequently repeated within a subgroup restricted to patients without monoclonal gammopathy.

All statistical computations were performed using R 4.0.3 (R Foundation for Statistical Computing, Vienna, Austria, www.R-project.org).

## Results

### Patient characteristics

A total of 122 patients, 44 female (36.1%) and 78 male (63.9%), were included. Mean ± SD patient age was 74.0 ± 11.2 years. Forty-nine (40.2%) patients were diagnosed with CA. Of those, 40 patients were diagnosed with wtATTR (32.8%), 6 with hATTR (4.9%) and 3 with AL amyloidosis (2.5%) (Table 1). Details on the applied diagnostic criteria are presented in Supplemental Table S1.

**Table 1:** Baseline Characteristics

Perugini Score	All	0	1	2	3	*p*-value (MANOVA)
Demographics	*n* = 122	*n* = 64	*n* = 18	*n* = 10	*n* = 30	
Female/Male	44/78	29/35	9/9	2/8	4/26	**0.006**
Mean Age (SD)	74.4(± 11.2)	72.0 (± 10.9)	74.4 (± 12.2)	75.7 (± 13.6)	77.4 (± 10.0)	0.621
Imaging						
Mean Injected Activity (SD)	522.4 (± 25.3)	520.5 (± 25.5)	528.8 (± 31.1)	516.2 (± 18.3)	524.2 (± 26.2)	0.480
Amyloidosis						
wtATTR (%)	40 (32.8%)	2 (3.1%)	2 (11.1%)	8 (80.0%)	28 (93.3%)	**< 0.0001**
hATTR (%)	6 (4.9%)	1 (1.6%)	1 (5.6%)	2 (20.0%)	2 (6.7%)	0.088
AL (%)	3 (2.5%)	3 (4.7%)	0 (0.0%)	0 (0.0%)	0 (0.0%)	0.121
No Amyloidosis (%)	73 (59.8%)	58 (90.6%)	15 (83.3%)	0 (0.0%)	0 (0.0%)	**< 0.0001**

wt/hATTR: wildtype/hereditary transthyretin amyloidosis, AL: lightchain amyloidosis

In late scintigraphy imaging, 64/122 patients had a Perugini score of 0 (52.5%), 18/122 a Perugini score of 1 (14.8%), 10/122 a Perugini score of 2 (8.2%), and 30/122 a Perugini score of 3 (24.6%). In MANOVA analysis, the fraction of male gender was significantly higher for higher Perugini scores (Tabl. 1) resembling the epidemiologic distribution of wtATTR amyloidosis [15]. Moreover, as expected, the fraction of wtATTR was significantly higher in patients with higher Perugini score (Tabl. 1). Image examples showing early and late scintigraphy images of a patient with Perugini score 0 and low visual cardiac tracer uptake at early timepoint as well as a patient with Perugini score 3 and high visual early cardiac uptake are presented in Fig. 1B.

### Correlation of early semiquantitative parameters and Perugini score

**Fig. 2:**
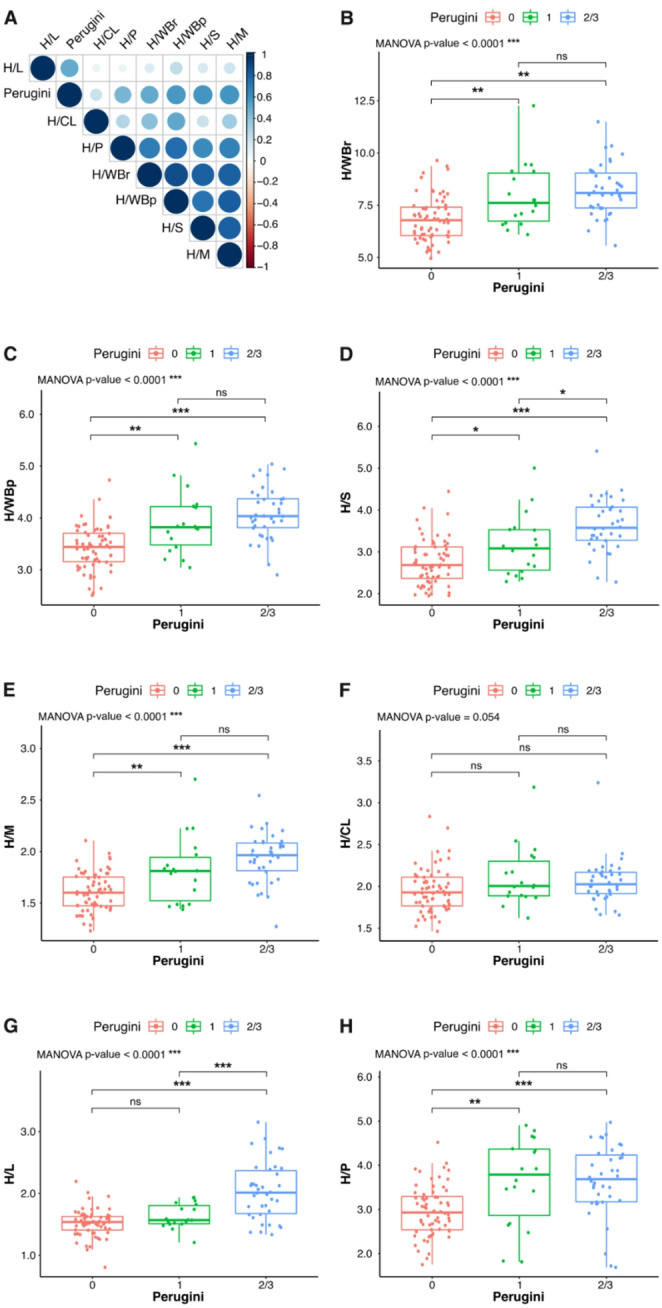
Correlation matrix (Spearman’s rho) of early-phase semi-quantitative indices. (**A**) Correlation matrix visualising Spearman’s rho between the different semiquantitative uptake ratios. (**B-H**) Boxplot representations of the semiquantitative uptake for different Perugini score groups (0 versus 1 versus 2/3) additionally indicating p-values for MANOVA and Scheffé analyses (*: p≤0.05, **: p≤0.01, ***: p≤0.001; ns: not significant). All semiquantitative ratios were calculated from uptake values derived from anterior images

Figure 2A presents the correlation analysis for uniplanar (anterior) projections. Of the investigated semiquantitative scores, all but H/CL showed a good correlation with the Perugini score. These parameters were also in good correlation with each other, except for H/L, which showed only weak correlations with the other indices. When evaluating arithmetic and geometric means from ventral and dorsal projections, the qualitative ranking of indices was preserved, whereas AUC values, optimal cut-offs, and sensitivity/specificity shifted modestly (Supplemental Tables S2–S7). Therefore, we focused the further main analysis on uniplanar anterior projections for clinical practicality.

Except for H/CL, all investigated semiquantitative scores were significantly higher in patients with increased Perugini score (Perugini 0 vs. Perugini 1 vs. Perugini 2/3). The post-hoc analysis showed significant differences between all three Perugini groups for H/S and significant differences between Perugini 0 and Perugini 2/3 as well as Perugini 1 and Perugini 2/3 (but not between Perugini 0 and Perugini 1) for H/L. The other parameters except for H/CL exhibited significant differences between Perugini 0 and Perugini 1 as well as Perugini 0 and Perugini 2/3 but not between Perugini 1 and Perugini 2/3. H/CL did not show any significant differences between Perugini groups. Boxplot representations are presented in Fig. 2B–H. Projection-dependent analyses based on arithmetic and geometric means showed consistent overall conclusions; detailed results are provided in Supplemental Tables S2–S7 to document robustness across projection strategies.

### Prediction of Perugini score ≥ 1

The highest performance in predicting a Perugini score ≥ 1 from early scintigraphy was observed with the H/WBp parameter, achieving an AUC of 0.82 and an accuracy of 76.7% (Table 2). This was closely followed by H/M (AUC 0.81, accuracy 77.5%) and H/S (AUC 0.80, accuracy 75.0%) (Table 2). Among all, H/CL demonstrated the greatest sensitivity (89.3%) but had the lowest specificity (35.9%), an AUC of 0.63, and an accuracy of 60.8% (Table 2). H/L, which exhibited minimal correlation with the other parameters, had an AUC of 0.77, an accuracy of 76.7%, and the highest specificity (93.8%) with a sensitivity of 57.1% (Table 2).

**Table 2:** Prediction of Perugini ≥ 1

Index	H/WBr	H/WBp	H/S	H/M	H/CL	H/L	H/*P*
Cut-off	7.09	3.78	3.13	1.81	1.85	1.79	3.42
AUC	0.78	0.82	0.80	0.81	0.63	0.77	0.76
Accuracy (%)	71.7	76.7	75.0	77.5	60.8	76.7	74.2
Sensitivity (%)	78.6	71.4	73.2	69.6	89.3	57.1	67.9
Specificity (%)	65.6	81.2	76.6	84.4	35.9	93.8	79.7
PPV (%)	66.7	76.9	73.2	79.6	54.9	88.9	74.5
NPV (%)	77.8	76.5	76.6	76.1	79.3	71.4	73.9

For semiquantitative parameters derived from the arithmetic and geometric means of ventral and dorsal projections, the overall diagnostic performance was slightly reduced but remained comparable; further details are available in **Supplemental Tables S2** and **S5**.

### Prediction of Perugini score ≥ 2

Best results for prediction of a Perugini score ≥ 2 from early scintigraphy images were achieved for H/S with an AUC of 0.83 and an accuracy of 78.3%, followed by H/M with an AUC of 0.82 and an accuracy of 81.7%, and H/L with an AUC of 0.82 and an accuracy of 83.3%. Of these, H/P showed the highest sensitivity of 86.8% but had a lower AUC of 0.71, an accuracy of 64.2%, and a specificity of 53.7%. H/L reached the highest specificity of 89.0% at a sensitivity of 71.1%.

Detailed results are presented in Table 3. For semiquantitative parameters calculated using the arithmetic and geometric means from ventral and dorsal projections, the qualitative conclusions were consistent with the primary anterior analysis, whereas AUC values, optimal cut-offs, and sensitivity/specificity showed modest shifts; details are given in Supplemental Tables S3 and S6.

**Table 3:** Prediction of Perugini ≥ 2

Index	H/WBr	H/WBp	H/S	H/M	H/CL	H/L	H/*P*
Cut-off	7.36	3.92	3.27	1.84	1.93	1.79	3.13
AUC	0.76	0.81	0.83	0.82	0.60	0.82	0.71
Accuracy (%)	70.0	80.8	78.3	81.7	55.8	83.3	64.2
Sensitivity (%)	78.9	68.4	76.3	73.7	73.7	71.1	86.8
Specificity (%)	65.9	86.6	79.3	85.4	47.6	89.0	53.7
PPV (%)	51.7	70.3	63.0	70.0	39.4	75.0	46.5
NPV (%)	87.1	85.5	87.8	87.5	79.6	86.9	89.8

### Prediction of Cardiac ATTR-Amyloidosis

The most effective predictor for ATTR-CA using early scintigraphy was H/L, with an AUC of 0.80 and an accuracy of 80.8%, followed by H/WBp (AUC 0.78, accuracy 79.2%) and H/S (AUC 0.78, accuracy 76.7%) (Table 4). H/L offered the best specificity (88.6%) alongside a sensitivity of 65.9%, whereas H/CL achieved the highest sensitivity (87.8%) but had the lowest specificity (30.4%), an AUC of 0.58, and an accuracy of 50.0% (Table 4). Parameters calculated from the average of ventral and dorsal projections did not substantially alter the findings; details are in **Supplemental Tables S4** and **S7**.

**Table 4:** Prediction of CA-ATTR

Index	H/WBr	H/WBp	H/S	H/M	H/CL	H/L	H/*P*
Cut-off	7.09	3.88	3.30	1.84	1.85	1.79	3.13
AUC	0.73	0.78	0.78	0.76	0.58	0.80	0.69
Accuracy (%)	65.8	79.2	76.7	79.2	50.0	80.8	63.3
Sensitivity (%)	80.5	65.9	68.3	68.3	87.8	65.9	82.9
Specificity (%)	58.2	86.1	81.0	84.8	30.4	88.6	53.2
PPV (%)	50.0	71.1	65.1	70.0	39.6	75.0	47.9
NPV (%)	85.2	82.9	83.1	83.8	82.8	83.3	85.7

### Combination of multiple semiquantitative parameters

As H/L showed only low correlation to the other semiquantitative parameters (Fig. 2), we tested a combination of H/L and the best performing scores H/WBp, H/S, and H/M to predict a Perugini score ≥ 1, a Perugini score ≥ 2, and CA. For comparison, we also analysed a combination of H/L and H/CL. As H/L in general showed higher specificity, whereas for H/WBp, H/S, H/M, and H/CL sensitivity was higher (Tables 2, 3 and 4), we used H/WBp, H/S, H/M, and H/CL as first cut-off parameters followed by H/L (Table 5).

**Table 5:** Combination of semiquantitative parameters

Prediction	Perugini ≥ 1				Perugini ≥ 2				Cardiac Amyloidosis			
Index 1	**H/WBp**	**H/S**	**H/M**	**H/CL**	**H/WBp**	**H/S**	**H/M**	**H/CL**	**H/WBp**	**H/S**	**H/M**	**H/CL**
Index 2	**H/L**	**H/L**	**H/L**	**H/L**	**H/L**	**H/L**	**H/L**	**H/L**	**H/L**	**H/L**	**H/L**	**H/L**
Cut-off 1	3.37	2.48	1.49	1.76	3.47	2.95	1.59	1.73	3.46	2.53	1.56	1.73
Cut-off 2	1.71	1.66	1.78	1.79	1.88	1.88	1.91	1.91	1.88	1.75	1.88	1.91
AUC	0.89	0.88	0.89	0.80	0.91	0.93	0.93	0.82	0.88	0.89	0.88	0.80
Accuracy (%)	78.3	78.3	75.8	74.2	82.5	84.2	81.7	80.8	80.8	83.3	80.8	78.3
Sensitivity (%)	57.1	60.7	53.6	51.8	55.3	57.9	52.6	55.3	53.7	63.4	56.1	51.2
Specificity (%)	96.9	93.8	95.3	93.8	95.1	96.3	95.1	92.7	94.9	93.7	93.7	92.4
PPV (%)	94.1	89.5	90.9	87.9	84.0	88.0	83.3	77.8	84.6	83.9	82.1	77.8
NPV (%)	72.1	73.2	70.1	69.6	82.1	83.2	81.2	81.7	79.8	83.2	80.4	78.5

For the combination of H/WBp, H/S, or H/M and H/L, accuracy and specificity results were substantially improved compared to single parameters, whereas sensitivity was slightly reduced. Accuracy and specificity were high to very high, whereas sensitivity was moderate. Overall, the results for H/WBp, H/S, or H/M in combination with H/L were comparable, with H/S reaching best results. To predict a Perugini score ≥ 1, AUC was 0.88, accuracy 78.3%, sensitivity 60.7%, and specificity 93.8%. To predict a Perugini score ≥ 2, AUC was 0.93, accuracy 84.2%, sensitivity 57.9%, and specificity 96.3%. To predict CA, AUC was 0.89, accuracy 83.3%, sensitivity 63.4%, and specificity 93.7%.

Additionally, requiring the absence of monoclonal protein for a prediction of CA analogue to proposed guidelines resulted in comparable overall performance, with an AUC of 0.89, accuracy of 83.2%, sensitivity of 64.9%, and specificity of 92.9% for the combination of H/S and H/L (details in **Tabl. 6**).

## Discussion

In this study, we evaluated the diagnostic value of semiquantitative parameters derived from an additional early acquisition (10 min after injection) in ^99m^Tc-DPD scintigraphy for prediction of the Perugini score and detection of CA. We were not able to confirm the excellent sensitivity and specificity results reported in a previous study using ^99m^Tc-HMDP [11], indicating that early imaging cannot replace standard imaging in clinical routine. However, we identified early H/L as a novel, highly specific semiquantitative measure and showed that a combination approach using two semiquantitative parameters can increase specificity. These concepts can easily be implemented in clinical routine imaging and may be beneficial to increase the accuracy of non-invasive imaging-based diagnosis of CA.

We found a H/M > 1.81 on early imaging to predict a Perugini score of ≥ 1 with a sensitivity of 69.6% and a specificity of 84.4% and a H/M > 1.84 on early imaging to predict ATTR-CA with a sensitivity of 68.3% and a specificity of 84.8%. These results do not reach the excellent accuracy described by Galat et al. for ^99m^Tc-HMDP who reported an early H/M > 1.115 to predict a Perugini score of ≥ 1 with a sensitivity of 100% and specificity of 97% and an early H/M > 1.20 to predict ATTR-CA- with a sensitivity of 100% and specificity of 100% [11]. A possible explanation the worse sensitivity and specificity and of variations in the optimal cut-off values are different tracer kinetics, differences in blood pool activity, and distinct early binding behaviours between ^99m^Tc-DPD and ^99m^Tc-HMDP. Moreover, calcium and plasma protein levels may affect the binding of these radiotracers to amyloid deposits in different manners [16]. This might also lead to different tracer binding kinetics with possible influences on the optimal time point to derive semi-quantitative scores with high predictive value. The American society of Nuclear Cardiology (ASNC) proposes H/CL for quantification of ^99m^Tc-PYP [13], in contrast to that our data showed medium high sensitivity with poor specificity of this metric in early images. H/CL is particularly sensitive to projection-dependent effects because the contralateral lung reference region can be influenced by attenuation/scatter differences and by overlapping with background uptake (e.g. mediastinal blood pool or skeletal activity), which is especially relevant in early-phase images with higher blood-pool activity. Averaging anterior and posterior views can therefore change the background counts in the lung ROI relative to myocardial counts, leading to different optimal cut-offs and to shifts in the sensitivity or specificity.

Furthermore, we identified early H/L as a novel, largely independent measure with the highest specificity among the evaluated early-imaging semiquantitative parameters for determining CA. The correlation of H/L with other established semiquantitative indices was low. To the best of our knowledge, this parameter has not yet been described for evaluation of CA from scintigraphy imaging using bone-seeking tracers. A possible pathophysiological explanation is that CA patients can exhibit strong cardiac and soft-tissue uptake but also reduced liver activity (photopenic liver), which may be explained by congestive hepatomegaly secondary to right-sided heart failure [5, 17]. Decreased early liver uptake could lead to an increased H/L. On the other hand, increased liver uptake can occur in CA patients with extra-cardiac organ involvement [5, 17], which could limit the applicability of H/L. Future evaluations are necessary to validate this measure in additional patient cohorts and to investigate its clinical value. Moreover, H/L derived at later imaging timepoints could be compared to early H/L.

Furthermore, right ventricular involvement is described in AL-CA in the later course of the disease, occurring later than LV involvement [18], whereas it is generally found in ATTR-CA. It would be of interest to evaluate H/L in a patient cohort with a larger fraction of AL-CA patients to investigate whether it can help differentiate between amyloidosis subforms. In this study, only three patients with AL-CA were included. Moreover, several studies report that right ventricular involvement is associated with worse survival in patients with CA [18, 19]. Assuming a possible relation between H/L and right ventricular function, future studies might investigate the prognostic potential of this parameter derived from early or late images.

Whereas sensitivity and specificity in early imaging were not sufficient to replace late imaging in clinical routine, early semi-quantitative scores might add additional value to late imaging. We were able to show that a combination of different early semi-quantitative measures can increase the accuracy in comparison to evaluation of one single measure. To the best of our knowledge this is the first description of combining different semi-quantitative measures to increase the accuracy. This might also hold for a combination of semi-quantitative scores derived from early- and late-timepoint imaging. A combination approach could also be beneficial to tackle the clinically problematic management of patients with a Perugini score of 1 which often requires endomyocardial biopsy [13].

Currently, different centres use various protocols with different imaging timepoints – mostly 1–3 h post injection depending on the scintigraphy tracer [7, 9] - and inclusion of either planar, Single Photon Emission Computed Tomography/Computed Tomography (SPECT/CT), or both imaging modalities [20]. In the context of recent position papers questioning if visual analysis is sufficient for imaging-based assessment of CA [21, 22], optimization of imaging protocols is of major relevance and future evaluations might investigate different combinations of imaging biomarkers. Analysis of semi-quantitative parameters at different time-points can be beneficial due to different tracer binding kinetics to cardiac amyloid and reference organs. For example, cardiac, soft-tissue, and bones show different, reciprocal uptake behaviour in ^99m^Tc-DPD scintigraphy over an interval of 3 h in patients with ATTR-CA [5]. This could be exploited by derivation of deriving semi-quantitative biomarkers at different time-points. An optimized imaging protocol may combine imaging results and imaging biomarkers at different optimized timepoints for highly accurate diagnosis of CA. In this context, early imaging is more comfortable for patients and can increase patient throughput [20]. On the other hand, late imaging may be beneficial for diagnosing extra-cardiac manifestations [5, 23].

An alternative to the evaluation of semi-quantitative scores from planar imaging is application of three-dimensional SPECT imaging. Quantitative cardiac SPECT/CT was introduced as a sophisticated method to calculate standardized uptake values (SUV) for a potentially more precise characterisation of myocardial uptake which can predict ATTR-CA with high accuracy [24–26]. Here, myocardial SUV can be time-dependent with a decrease between 1 h and 3 h after tracer injection [27] and retention indices may be calculated as additional imaging biomarkers. Scully et al. reported that SUV retention index and cardiac SUVpeak outperforms planar evaluation in differentiation of Perugini Score 2 and 3 [28]. If combinations of semi-quantitative markers derived from different time-points may show comparable results to SPECT, these might be used to derive less laborious imaging protocols to increase patient comfort. Moreover, they can be beneficial in centres that are not equipped with SPECT cameras. On the other hand, combinations of different SPECT and planar imaging biomarkers might be even more accurate and SPECT can, additionally, localize the cardiac uptake, for example in the septum [29].

The study has several limitations. First, the cohort of patients with diagnosed CA consisted mostly of patients with ATTR-CA, whereas only three patients with AL-CA were included; therefore, the diagnostic precision of early semiquantitative imaging biomarkers for differentiating ATTR-CA from AL-CA could not be evaluated. In addition, potential differences in tracer kinetics and background/organ uptake between ATTR-CA and AL-CA may particularly affect liver-related indices such as H/L; thus, the applicability of the proposed early H/L threshold to AL-CA remains uncertain based on the present data. Second, imaging data were acquired at a single centre using exclusively ^99m^Tc-DPD. Given the discrepancy with previous results described for ^99m^Tc-HMDP [14], future studies are necessary for a direct comparison of available bone-seeking tracers. Third, early planar ROIs reflect whole-heart uptake including blood-pool contribution, and inter- and intra-observer reproducibility of ROI-based measurements was not assessed, which may limit reproducibility and generalizability due to ROI placement variability. Finally, the proposed thresholds were derived from this retrospective cohort and might be optimistic, therefore external prospective validation is warranted.

### New Knowledge Gained

In contrast to previous results in ^99m^Tc-HMDP, evaluation of semiquantitative biomarkers derived from early ^99m^Tc-DPD imaging revealed limited accuracy, which is not sufficient to replace late imaging in clinical routine for diagnosing CA. However, early H/L was identified as a novel, largely independent, and highly specific imaging biomarker that can be added as an additional parameter to imaging protocols. This is the first study to show that a combination of different semiquantitative parameters can be used to increase the accuracy and precision for imaging-based diagnosis of CA. Table. 6

**Table 6:** Combination of semiquantitative parameters and absence of monoclonal protein

Prediction	Perugini ≥ 1				Perugini ≥ 2				Cardiac Amyloidosis			
Index 1	**H/WBp**	**H/S**	**H/M**	**H/CL**	**H/WBp**	**H/S**	**H/M**	**H/CL**	**H/WBp**	**H/S**	**H/M**	**H/CL**
Index 2	**H/L**	**H/L**	**H/L**	**H/L**	**H/L**	**H/L**	**H/L**	**H/L**	**H/L**	**H/L**	**H/L**	**H/L**
Cut-off 1	3.37	2.48	1.49	1.85	3.47	2.95	1.68	1.85	3.23	2.42	1.47	1.73
Cut-off 2	1.71	1.7	1.79	1.75	1.88	1.91	1.79	1.79	1.79	1.79	1.88	1.88
AUC	0.88	0.88	0.89	0.8	0.91	0.93	0.94	0.85	0.88	0.89	0.89	0.83
Accuracy (%)	78.5	78.5	75.7	75.7	84.1	85.0	86.9	85.0	83.2	83.2	81.3	79.4
Sensitivity (%)	58.0	58.0	54.0	54.0	58.8	58.8	67.6	67.6	64.9	64.9	59.5	56.8
Specificity (%)	96.5	96.5	94.7	94.7	95.9	97.3	95.9	93.2	92.9	92.9	92.9	91.4
PPV (%)	93.5	93.5	90.0	90.0	87.0	90.9	88.5	82.1	82.8	82.8	81.5	77.8
NPV (%)	72.4	72.4	70.1	70.1	83.3	83.5	86.4	86.1	83.3	83.3	81.3	80.0

## Conclusion

We investigated the diagnostic accuracy of different semiquantitative parameters derived from early ^99m^Tc-DPD scintigraphy acquired 10 min after tracer injection for diagnosis of CA. We were not able to confirm the excellent sensitivity and specificity results reported in a previous study for ^99m^Tc-HMDP. Accuracy results of about 80% for diagnosis of CA are not sufficient to replace late imaging in clinical routine. However, we identified early H/L as a novel, largely independent, and highly specific imaging biomarker. Moreover, we showed that accuracy and specificity can be improved by adding early H/L to other early semiquantitative parameters. Combinations of different early and late semiquantitative measures might be used in future studies to derive an optimized imaging protocol.

Figure 2. Correlation matrix (Spearman’s rho) of early-phase semi-quantitative indices. (**A**) Correlation matrix visualising Spearman’s rho between the different semiquantitative uptake ratios. (**B-H**) Boxplot representations of the semiquantitative uptake for different Perugini score groups (0 versus 1 versus 2/3) additionally indicating p-values for MANOVA and Scheffé analyses (*: *p* ≤ 0.05, **: *p* ≤ 0.01, ***: *p* ≤ 0.001; ns: not significant). All semiquantitative ratios were calculated from uptake values derived from anterior images.

## Supplementary Information

Below is the link to the electronic supplementary material.

Supplementary material 1 (DOCX 25.5 kb)

## Abbreviations

AL: Light chain amyloidosis.
ATTR: Transthyretin amyloidosis.
AUC: Area Under Curve
CA: Cardiac amyloidosis.
H/WBr: Heart/whole-body rectangular ratio.
H/WBp: Heart/whole-body profile ratio.
H/S: Heart/Skull ratio
H/M: Heart/Mediastinum ratio.
H/CL: Heart/Contralateral Lung ratio.
H/L: Heart/Liver ratio.
H/P: Heart/Pelvis ratio.
HFpEF: Heart failure with preserved ejection fraction.
NPV: Negative predictive value.
PPV: Positive predictive value.
ROC: Receiver operating characteristic.
ROI: Region-of-interests.
SPECT/CT: Single Photon Emission Computed Tomography/Computed Tomograpy.
^99m^Tc-DPD ^99m^Tc: diphosphono-propanodicarboxylic acid.
^99m^Tc-HMDP ^99m^: Tc-hydroxymethylene-diphosphonate acid.
^99m^Tc-PYP ^99m^Tc: pyrophosphate.
