# Supplementary material for: Semi-quantitative evaluation of early phase 99mTc-DPD scintigraphy in patients with suspected cardiac amyloidosis
Source: Ann Nucl Med. 2026 Apr 16;40(8):949–60. doi: 10.1007/s12149-026-02209-w (PMC13388646; doi:10.1007/s12149-026-02209-w)
Supplement: Supplementary file 1 — Supplementary material 1 (DOCX 25.5 kb) [file 12149_2026_2209_MOESM1_ESM.docx]

#### Supplemental Table S1 Details of Diagnostic Criteria

| Diagnosis/  Applied Diagnostic Criteria | wtATTR | hATTR | AL | Total  (n = 49) |
| --- | --- | --- | --- | --- |
| Endomyocardial biopsy | 20 (40.8%) | – | 1 (2.0%) | 21 (42.9%) |
| Extracardiac tissue biopsy + Perugini >2 + absence of monoclonal protein | 4 (8.2%) | – | – | 4 (8.2%) |
| Extracardiac tissue biopsy + pathological imaging*  + absence of monoclonal protein | 2 (4.1%) | 2 (4.1%) | – | 4 (8.2%) |
| Extracardiac tissue biopsy + pathological imaging* | – | – | 2 (4.1%) | 2 (4.1%) |
| Perugini >2 + absence of monoclonal protein | 14 (28.6%) | 4 (8.2%) | – | 18 (36.7%) |

wt/hATTR: wildtype/hereditary transthyretin amyloidosis, AL: lightchain amyloidosis.

*Pathological findings in magnetic resonance imaging or echocardiography.

#### Supplemental Table S2 Prediction of Perugini ≥1 for arithmetic mean

| Index | H/WBr | H/WBp | H/S | H/M | H/CL | H/L | H/P |
| --- | --- | --- | --- | --- | --- | --- | --- |
| Cut-off | 6.84 | 3.60 | 3.22 | 1.78 | 1.92 | 1.59 | 2.86 |
| AUC | 0.75 | 0.79 | 0.81 | 0.77 | 0.61 | 0.77 | 0.72 |
| Accuracy (%) | 70.0 | 74.2 | 75.8 | 75.8 | 61.7 | 75.8 | 70.8 |
| Sensitivity (%) | 75.0 | 62.5 | 64.3 | 73.2 | 28.6 | 58.9 | 46.4 |
| Specificity (%) | 65.6 | 84.4 | 85.9 | 78.1 | 90.6 | 90.6 | 92.2 |
| PPV (%) | 65.6 | 77.8 | 80.0 | 74.5 | 72.7 | 84.6 | 83.9 |
| NPV (%) | 75.0 | 72.0 | 73.3 | 76.9 | 59.2 | 71.6 | 66.3 |

#### Supplemental Table S3 Prediction of Perugini ≥2 for arithmetic mean

| Index | H/WBr | H/WBp | H/S | H/M | H/CL | H/L | H/P |
| --- | --- | --- | --- | --- | --- | --- | --- |
| Cut-off | 6.47 | 3.56 | 3.05 | 1.78 | 1.92 | 1.62 | 2.60 |
| AUC | 0.71 | 0.76 | 0.83 | 0.77 | 0.54 | 0.81 | 0.64 |
| Accuracy (%) | 60.8 | 72.5 | 73.3 | 75.0 | 66.7 | 84.2 | 65.0 |
| Sensitivity (%) | 86.8 | 76.3 | 86.8 | 81.6 | 26.3 | 73.7 | 60.5 |
| Specificity (%) | 48.8 | 70.7 | 67.1 | 72.0 | 85.4 | 89.0 | 67.1 |
| PPV (%) | 44.0 | 54.7 | 55.0 | 57.4 | 45.5 | 75.7 | 46.0 |
| NPV (%) | 88.9 | 86.6 | 91.7 | 89.4 | 71.4 | 88.0 | 78.6 |

#### Supplemental Table S4 Prediction of CA-ATTR for arithmetic mean

| Index | H/WBr | H/WBp | H/S | H/M | H/CL | H/L | H/P |
| --- | --- | --- | --- | --- | --- | --- | --- |
| Cut-off | 6.47 | 3.56 | 3.41 | 1.84 | 1.72 | 1.62 | 2.64 |
| AUC | 0.68 | 0.73 | 0.78 | 0.72 | 0.49 | 0.80 | 0.62 |
| Accuracy (%) | 60.0 | 71.7 | 78.3 | 76.7 | 58.3 | 81.7 | 65.8 |
| Sensitivity (%) | 82.9 | 73.2 | 58.5 | 63.4 | 64.6 | 68.3 | 53.7 |
| Specificity (%) | 48.1 | 70.9 | 88.6 | 83.5 | 46.3 | 88.6 | 72.2 |
| PPV (%) | 45.3 | 56.6 | 72.7 | 66.7 | 69.9 | 75.7 | 50.0 |
| NPV (%) | 84.4 | 83.6 | 80.5 | 81.5 | 40.4 | 84.3 | 75.0 |

#### Supplemental Table S5 Prediction of Perugini ≥1 for geometric mean

| Index | H/WBr | H/WBp | H/S | H/M | H/CL | H/L | H/P |
| --- | --- | --- | --- | --- | --- | --- | --- |
| Cut-off | 6.42 | 3.36 | 3.20 | 1.78 | 1.91 | 1.56 | 2.58 |
| AUC | 0.74 | 0.78 | 0.81 | 0.75 | 0.58 | 0.76 | 0.72 |
| Accuracy (%) | 69.2 | 71.7 | 75.8 | 71.7 | 60.8 | 75.8 | 70.0 |
| Sensitivity (%) | 83.9 | 82.1 | 64.3 | 69.6 | 28.6 | 58.9 | 60.7 |
| Specificity (%) | 56.2 | 62.5 | 85.9 | 73.4 | 89.1 | 90.6 | 78.1 |
| PPV (%) | 62.7 | 65.7 | 80.0 | 69.6 | 69.6 | 84.6 | 70.8 |
| NPV (%) | 80.0 | 80.0 | 73.3 | 73.4 | 58.8 | 71.6 | 69.4 |

#### Supplemental Table S6 Prediction of Perugini ≥2 for geometric mean

| Index | H/WBr | H/WBp | H/S | H/M | H/CL | H/L | H/P |
| --- | --- | --- | --- | --- | --- | --- | --- |
| Cut-off | 6.42 | 3.42 | 3.44 | 1.83 | 1.75 | 1.62 | 2.58 |
| AUC | 0.70 | 0.74 | 0.82 | 0.75 | 0.49 | 0.80 | 0.63 |
| Accuracy (%) | 60.8 | 69.2 | 82.5 | 76.7 | 54.2 | 85.0 | 66.7 |
| Sensitivity (%) | 86.8 | 81.6 | 63.2 | 68.4 | 54.9 | 73.7 | 60.5 |
| Specificity (%) | 48.8 | 63.4 | 91.5 | 80.5 | 52.6 | 90.2 | 69.5 |
| PPV (%) | 44.0 | 50.8 | 77.4 | 61.9 | 71.4 | 77.8 | 47.9 |
| NPV (%) | 88.9 | 88.1 | 84.3 | 84.6 | 35.1 | 88.1 | 79.2 |

#### Supplemental Table S7 Prediction of CA-ATTR for geometric mean

| Index | H/WBr | H/WBp | H/S | H/M | H/CL | H/L | H/P |
| --- | --- | --- | --- | --- | --- | --- | --- |
| Cut-off | 6.42 | 3.42 | 3.44 | 1.83 | 1.73 | 1.62 | 2.58 |
| AUC | 0.67 | 0.72 | 0.78 | 0.70 | 0.52 | 0.79 | 0.62 |
| Accuracy (%) | 60.0 | 68.3 | 80.0 | 74.2 | 58.3 | 82.5 | 65.8 |
| Sensitivity (%) | 82.9 | 78.0 | 58.5 | 63.4 | 62.0 | 68.3 | 58.5 |
| Specificity (%) | 48.1 | 63.3 | 91.1 | 79.7 | 51.2 | 89.9 | 69.6 |
| PPV (%) | 45.3 | 52.5 | 77.4 | 61.9 | 71.0 | 77.8 | 50.0 |
| NPV (%) | 84.4 | 84.7 | 80.9 | 80.8 | 41.2 | 84.5 | 76.4 |
